# Severe aggravation and possible triggering of pemphigus vulgaris following COVID-19 vaccination: report of two cases^☆^

**DOI:** 10.1016/j.abd.2023.04.012

**Published:** 2024-06-03

**Authors:** Kinuko Irie, Toshiyuki Yamamoto

**Affiliations:** Department of Dermatology, Fukushima Medical University, Fukushima, Japan

Dear Editor,

Pemphigus vulgaris (PV) is a severe autoimmune blistering dermatosis. Genetic, malignant, and drug-induced PV triggers have been reported. Here we report two cases of patients who had severe aggravation or exacerbation of PV after COVID-19 vaccination.

Case 1: A 60-year-old man with a two months history of painful erosion on the oral mucosa, which was treated by an otolaryngologist and an internist, received the second dose of the COVID-19 vaccine (Comirnaty®). One week later, erythema and erosions appeared at the vaccination site on his left arm. Subsequently, he developed erythema and erosions on his trunk and was referred to our department one month after the vaccination. Clinical examination showed the presence of post-bullous erosions, especially on the trunk, scalp, and left arm (Fig. 1A). He also had multiple erosions on the buccal mucosa (Fig. 1B). A skin biopsy showed acantholysis within the lower epidermal layers, and the presence of dense lymphocytic and eosinophilic dermal infiltrates (Fig. 2A). Direct immunofluorescence (DIF) revealed intercellular deposition of IgG and C3 in the epidermal cells (Fig. 2B). Serum levels of anti-Desmoglein (Dsg)-1 antibodies (120 U/mL, normal < 3 U/mL) and anti-Dsg-3 antibodies (262 U/mL, normal < 3 U/mL) were elevated. Oral prednisolone (50 mg/day (1 mg/kg/day)) was started; however, the response was poor and thus methylprednisolone pulse (1000 mg/day for consecutive three days), plasma exchange, and methotrexate (6 mg/week) were added. After obtaining remission, he received third and fourth dose of the COVID-19 vaccination without recurrence.

**Figure 1:**
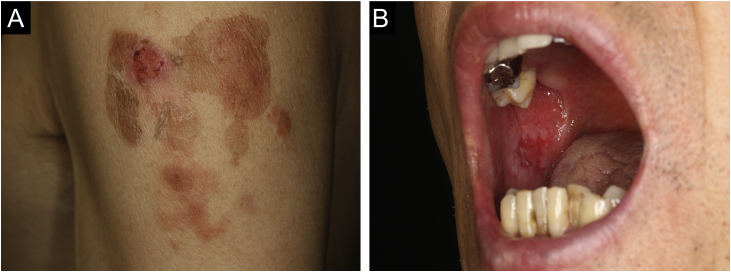
(A) Crusted plaque and erythema at the vaccination site on the left upper arm. (B) Erosions of the oral mucosa.

**Figure 2:**
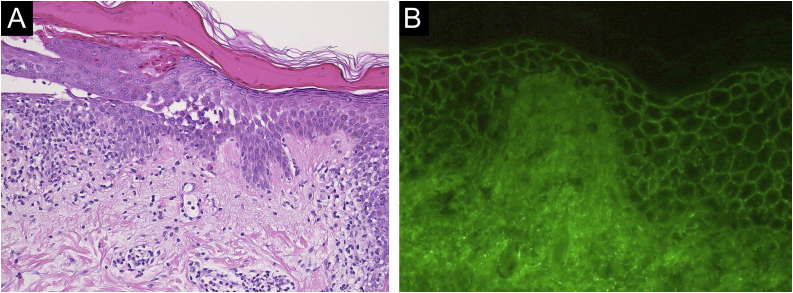
(A) Histopathological findings showing acantholysis within the lower epidermal layers, and the presence of dense lymphocytic and eosinophilic dermal infiltrates (×40). (B) Direct immunofluorescence revealed intercellular deposition of IgG in the epidermal cells (×40).

Case 2: A 69-year-old woman received the third dose of the COVID-19 vaccine (Spikevax®), and at about the same time, she experienced a scald injury on her right arm. Subsequently, she developed erosions on her extremities and trunk, which gradually increased in number, and was referred to our department three months after the vaccination. Clinical examination showed extensive erythema, flaccid blisters and post-bullous erosions on the trunk (Fig. 3). She also had multiple erosions on the oral mucosa. A skin biopsy from her abdomen revealed acantholysis within the lower epidermal layers, and the presence of dense lymphocytic and neutrophilic dermal infiltrates (Fig. 4A). DIF revealed intercellular deposition of IgG (Fig. 4B) and C3 in the lower epidermal cells. Serum levels of anti-Dsg-3 antibodies were high (8360 U/mL, normal < 3 U/mL), whereas those of anti-Dsg-1 antibodies were normal. Treatment with oral prednisolone (45 mg/day [1 mg/kg/day]), methylprednisolone pulse therapy (1000 mg/day for consecutive three days), and azathioprine (100 mg/day) resulted in complete epithelialization of erosions after 5 weeks of treatment.

**Figure 3:**
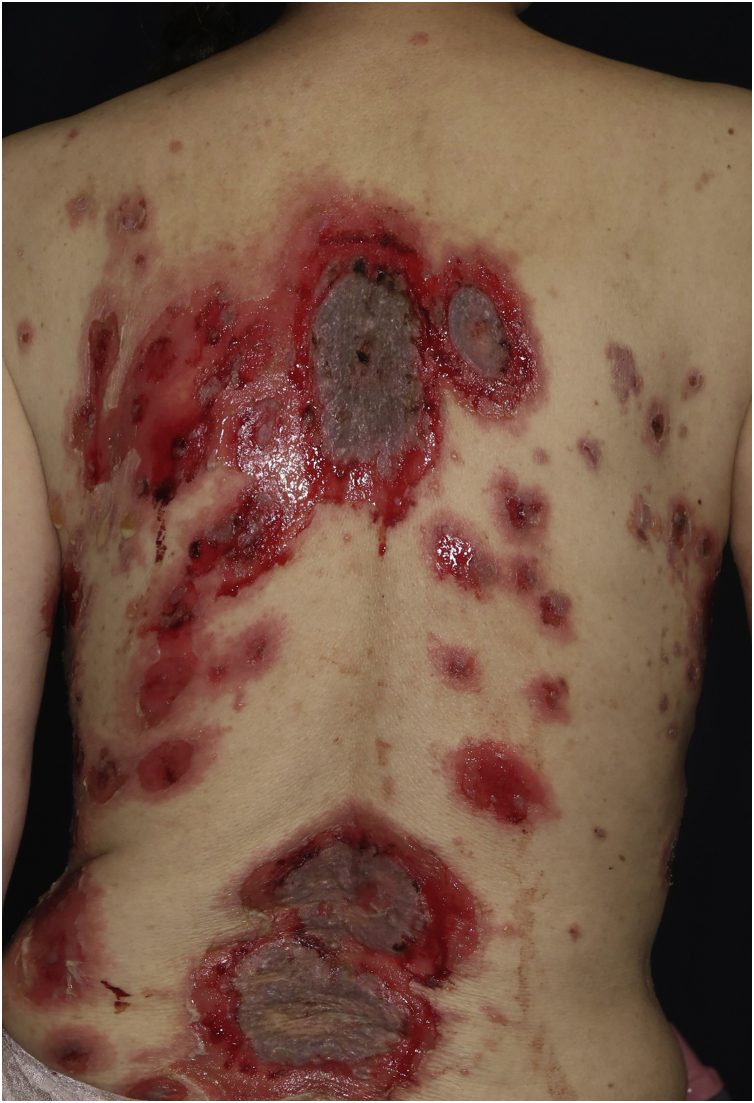
Extensive erythema, flaccid blisters and post-bullous erosions on the trunk.

**Figure 4:**
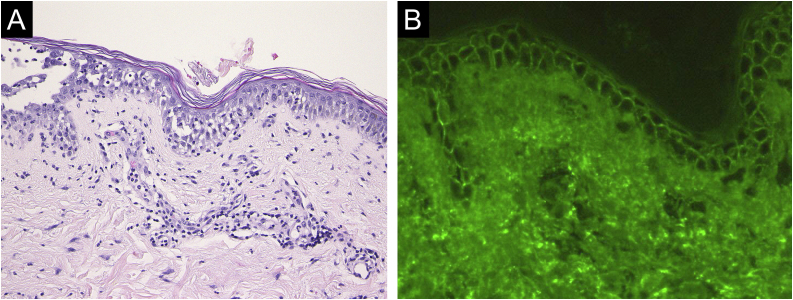
(A) Histopathological findings showing acantholysis within the lower epidermal layers, and the presence of dense lymphocytic and neutrophilic dermal infiltrates (×40). (B) Direct immunofluorescence revealed intercellular deposition of IgG in the lower epidermal cells (×40).

New onset or exacerbation of PV triggered by vaccinations or viral infections have been reported.^1^, ^2^, ^3^, ^4^, ^5^, ^6^, ^7^, ^8^, ^9^ There have been reported cases of PV induction or exacerbation following vaccination against influenza, rabies, hepatitis B, tetanus and diphtheria.^1^ In addition, cases of induction or exacerbation of autoimmune bullous diseases, psoriasis, lichen planus, dermatomyositis, and SLE, following COVID-19 vaccination have recently been reported.^10^ Activation of innate immunity due to the vaccine is thought to be the cause of exacerbation or development of skin symptoms. BNT162b2 injection induces activation of T-cells and B-cells, and after injection, CD4+ and CD8+ T-cells increase with production of IFN-γ and IL-2.^2^ It has been suggested that COVID-19 vaccination contributes to the production of cytokines like IL-4, IL-17, and IL-21 that play important roles in autoimmune bullous diseases such as PV.^3^ Vaccinations also activate B-cells, leading to increased antibody production.^4^ The reported cases^1^, ^2^, ^3^, ^4^, ^5^, ^6^, ^7^, ^8^, ^9^ of PV that developed *de novo* or deteriorated following COVID-19 vaccination are summarized in Table 1. PV developed a median of 7 (range 1–30) days each after the first, second, and third vaccinations. In contrast, the median time for cases of exacerbations was 3 days (range 3‒14), which is a significantly shorter period of time than the onset cases. However, there are two cases who were able to receive additional vaccinations after undergoing enhanced treatment for PV without flare-up of the disease. One of our cases also allowed for additional COVID-19 vaccinations without worsening the disease. The COVID-19 vaccine can certainly exacerbate PV in very rare cases, but even if exacerbation occurs, the vaccine can be safely administered in PV patients whose disease is well-controlled. Since vaccination is a necessary procedure to prevent aggravation of COVID-19 in immunosuppressed patients, the rare cases of progression of PV should not discourage the vaccination of patients with PV.

**Table 1:** Reported cases of pemphigus vulgaris triggered by, or exacerbated following, COVID-19 vaccination.

Nº	Author	Age (years)	Sex	Vaccine	Location of bullous lesion	Dose	New onset/Flare	Time-to-onset (days)	Dsg1/Dsg3	Treatment	Booster vaccination
1	Singh et al.^1^	44	M	ChAdOx1 nCov-19	Oral mucosa trunk, face, neck, extremities	2^nd^	New	7	NA/+	OC/IVC/	No
										IVIg/AZP/	
2	Thongprasom et al.^1^	38	F	AZD1222	Oral mucosa	1^st^	New	7	NA	TC	No
3	Koutlas et al.^1^	60	M	mRNA-1273	Oral mucosa	2^nd^	New	7	-/-	OC/RTX	No
4	Knechtl et al.^1^	89	M	BNT162b2	Oral mucosa, trunk, back, left arm	2^nd^	New	30	+/+	OC/RTX	No
5	Damiani et al.^1^	40	M	mRNA-1273	Back, upper limbs	1^st^	Flare	3	NA	OC increased/MMF	Received after treatment for PV/No change in PV condition.
6	Damiani et al.^1^	80	M	BNT162b2	Back	1^st^	Flare	3	NA	OC	Received after treatment for PV/No change in PV condition.
7	Solimani et al.^2^	40	F	BNT162b2	Oral mucosa, trunk, back	1^st^/2^nd^	New/Flare	5/3	+/+	OC/AZP	Received before treatment for PV/PV lesions worsened.
8	Shakoei et al.^3^	28	F	BBIBP-CorV	N/A	1^st^	Flare	14	NA	OC/RTX	No
9	Shakoei et al.^3^	30	F	BBIBP-CorV	Oral mucosa	1^st^	New	16	NA	OC/RTX	No
10	Corrá et al.^4^	61	F	BNT162b2	Face, trunk	3^rd^	New	3	+/+	OC	No
11	Corrá et al.^4^	73	F	BNT162b2	Oral mucosa	3^rd^	New	30	NA/+	OC/RTX	No
12	Corrá et al.^4^	63	F	ChAdOx1 nCov-19	Oral mucosa, face, trunk	1^st^/2^nd^	New/Flare	28/4	+/+	OC/RTX	Received before treatment for PV/PV lesions worsened.
13	Calabria et al.^5^	60	F	BNT162b2	Oral mucosa, oropharynx mucosa	2^nd^	New	7	-/+	OC/RTX	
14	Akoglu^5^	69	F	ChAdOx1 nCov-19	Oral mucosa, head, limbs	1^st^	New	7	+/+	MTX	No
15	Agharbi et al.^6^	72	M	BNT162b2	Oral mucosa, trunk, head, neck, extremities	2^nd^	New	7	+/+	OC/AZP	No
16	Hali et al.^7^	58	F	BNT162b2	Oral and genital mucosa, trunk, face, neck, extremities	1^st^	New	30	NA	OC	Scheduled intake.
17	Norimatsu et al.^8^	86	M	BNT162b2	left arm, face, lumbus	2^nd^	New	1	+/+	OC/IVC	No
18	Saffarian et al.^9^	76	F	BBIBP-CorV	Oral and genital mucosa, trunk, head	2^nd^	New	30	-/-	OC/RTX	No
19	Our case 1	60	M	BNT162b2 (1^st^, 2^nd^)	Oral mucosa, scalp, trunk, left arm	2^nd^	Flare	7	+/+	OC/IVC/PE/MTX	Received after treatment for PV/No change in PV condition.
20	Our case 2	69	F	BNT162b2 (1^st^, 2^nd^), mRNA-1273 (3^rd^)	Oral mucosa, trunk, back	3^rd^	New or Flare	NA	-/+	OC/IVC/AZP	No

NA, Noavailable; DSG1, Antibody anti-Desmoglein 1; DSG3, Antibody anti-Desmoglein 3; TC, Topical Corticosteroids; OC, Oral Corticosteroids; IVC, Intravenous Corticosteroids; RTX, Rituximab; MTX, Methotrexate; AZP, Azathioprine; MMF, Mycophenolate Mofetil; IVIg, Intravenous high-dose Immunoglobulin therapy; PE, Plasma Exchange; PV, Pemphigus Vulgaris.

## Financial support

None declared.

## Authors’ contributions

Kinuko Irie: Critical literature review; Data collection, analysis and interpretation; preparation and writing of the manuscript; statistical analysis; study conception and planning; approval of the final version of the manuscript.

Toshiyuki Yamamoto: Study conception and planning; manuscript critical review; approval of the final version of the manuscript.

## Conflicts of interest

None declared.
